# Assessment of the Attitude and Knowledge of the Principles and Practices of Orthodontic Treatment Among the Non-orthodontic Specialists and General Practitioner Dentists

**DOI:** 10.31729/jnma.3674

**Published:** 2018-08-31

**Authors:** Deepika Kapoor, Sandeep Bhatia, Deepanshu Garg

**Affiliations:** 1 Department of Pedodontics, College of Medical Sciences and Teaching Hospital, Bharatpur, Nepal; 2 Department of Orthodontics and Dentofacial Orthopedics, Daswani Dental College, Kota, Rajasthan; 3 Department of Oral Medicine and Radiology, College of Medical Sciences and Teaching Hospital, Bharatpur, Nepal

**Keywords:** *assessment*, *attitude*, *knowledge*, *orthodontic treatment*

## Abstract

**Introduction:**

General practitioner dentists and non-orthodontic specialties ought to have the knowledge of the basic principles and practices of orthodontics in order to educate the patients, diagnose their problems correctly and for proper referral. The objective of the present study is to assess the attitude and knowledge of the general practitioner dentists and non-orthodontic specialists towards the basic principles and practices of orthodontics.

**Methods:**

This study was performed by presenting a closed questionnaire to a total of 78 participants out of which 46 were general practitioners and 32 were non-orthodontic specialists. A questionnaire consisting of a total of 21 questions was distributed and each question was allocated 0.5 marks for correct response whereas no deduction for wrong answer.

**Results:**

In this present study, the total mean score of the evaluation of the questionnaire came out for general practitioner dentist and the non-orthodontic dental specialists was 13.92 and 16.69 respectively. The present study showed a statistically highly significant knowledge and attitude difference between Group A and Group B ( P<0.001).

**Conclusions:**

This study shows a need for a increased clinically oriented education in the undergraduate courses and a multi-disciplinary inter department seminar presentations and forums set up for the post graduation courses for them to understand the scope of each other's specialties.

## INTRODUCTION

Malocclusion has a horizon of causes but the outcomes are quite common including unaesthetic appearance, patient discomfort, gingival and periodontal problems, difficulty in chewing, speech problems, and etc.^1^ So, a multidisciplinary approach of patient education is required for them to understand the need of orthodontic treatment.

Thus, general practitioner dentists and non-orthodontic specialties ought to have the knowledge of the basic principles and practices of orthodontics in order to educate the patients.^2^ Many times patient might present with a chief complaint that he/she would not be able to correlate with an underlying malocclusion. In that case, it is imperative for the dentist to identify and diagnose the chief cause which may be an orthodontic cause and then plan a proper referral.^3^

This study is done with an objective to assess the attitude and knowledge of the general practitioner dentists and non-orthodontic specialists towards the basic principles and practices of orthodontics.

## METHODS

The present cross-sectional descriptive study was conducted at College of Medical Sciences and Teaching Hospital, Bharatpur, Nepal and on the passed out students of COMS-TH, Nepal who are currently general practitioner dentists. The study was initiated with the approval of the proposal of study to COMSTH-IRC in May 2018 and was completed with the preparation of manuscript in June 2018. An informed consent was taken after explaining all the required details, the importance and the possible implications of the study through personal contact and e-mail. Those who did not give consent were excluded from the present study. To main the utmost confidentiality, the personal details are not disclosed or circulated anywhere except for between the researchers and so the names of the participants are not disclosed in the final report.

The inclusion criteria included general practitioner dentists who have completed their bachelor degree from COMS-TH, Bharatpur and the dental specialists other than the orthodontists in Bharatpur. Exclusion criteria included dental practitioners who are not currently practicing anywhere.

The study participants were enrolled using a convenience sampling technique and standard questionnaire from a previous study conducted by Sastri MR et al^2^ was used.

A sample size of 78 dentists was taken using the following formula:


n = (Za2)(P) (Q)/d2

Where, n=required sample size, Za=Variate corresponding to desired reliability level (1.96 for 95% reliability), P=Estimated proportion in the population (5% for this study)^2^, Q=100-P (if P is in %) and d=Maximum tolerable error (5%). According to this formula, the minimum required sample size was 70.

The sample was further divided into two groups. Group A consisted of 46 general practitioner dentists with a bachelor degree and the Group B included 32 dental specialists with masters in other specialties than orthodontics.

The questionnaire survey included a total number of 21 questions out of which 13 questions were formulated to study the knowledge where each correct answer was given a score of 1 and each incorrect answer was scored zero. The questionnaire was derived from a previous study and the questions mainly included: the right age for treatment, facial appearance, proclined teeth, functional therapy, mixed dentition treatment, habits, anchorage, etc. The questionnaire also included another 8 questions to study the attitude towards orthodontic treatment. Participants were recruited through personal contact and e-mail. The findings were put in a performa developed for this particular study and was then entered into Microsoft Excel. The stastistical analysis was performed using SPSS software with the help of Student's t-test and the results were then analyzed.

## RESULTS

**Figure 1.**
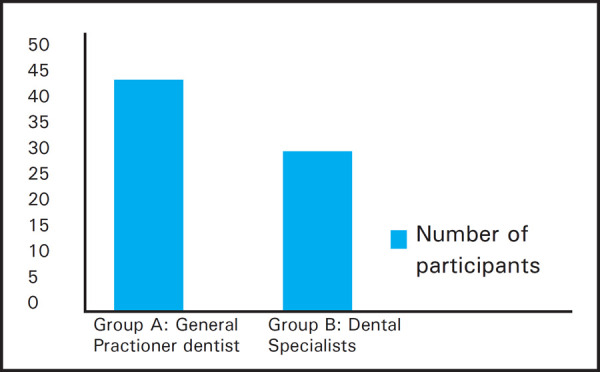
Total Number of participants and group division of the study.

**Figure 2.**
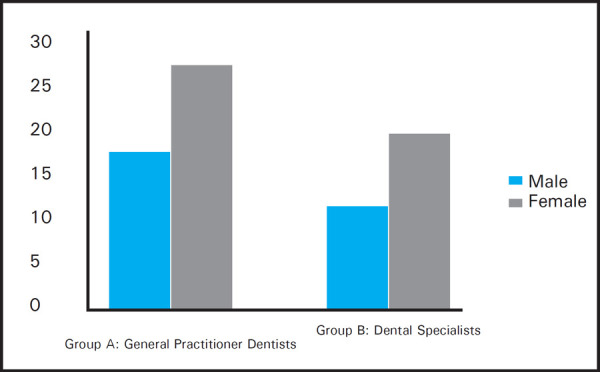
Sex distribution of the participants.

The study population included 78 total participants out of which 32 were dental specialists and 46 were general practitioner dentists (Figure 1). Out of these, 48 were females and 30 male dentists (Figure 2).

In this present study, the total mean score of the evaluation of the questionnaire came out for general practitioner dentist and the non-orthodontic dental specialists was 13.92 and 16.69 respectively (Table 1).

**Table 1:** The mean score of the knowledge and attitude of the sample.

GROUP	Knowledge	Attitude	Total Score
General practitioner Dentist	8.878	5.042	13.92
Specialist Dentist	10.245	6.445	16.69

The present study showed a statistically highly significant knowledge and attitude difference between Group A and Group B ( P<0.001) (Table 2).

**Table 2:** Comparison of the scores of knowledge and attitude of the two groups using Student's t-test.

Questionnare	Group	Mean±SD	t value	Signifinace
Knowledge	Group A	8.878	3.9906	P<0001**
	Group B	10.245		
Attitude	Group A	5.042	5.0054	P<0.001**
	Group B	6.445		

## DISCUSSION

Malocclusion is a common dental problem running at second number after the dental caries worldwide.^4^ Dental malocclusion can have a plethora of implications and the most common being on facial aesthetics. Many associated problems also include periodontal problems, difficulty in mastication, speech, swallowing, TMJ problems, associated habit development, etc.^5^ Many a times, patients come to the dentists with one of the associated problems as the chief complaint and at that time, it is imperative for the dentist to recognize the key cause and understand the need for orthodontic treatment.^6^

The present study focuses on the knowledge of dental practitioners both general and non-orthodontic specialties towards the basic principles and practices of orthodontic treatment. This study highlights a highly significant difference in the knowledge as well as attitude of general practitioner dentists and the specialists towards the orthodontic treatment. Similar results were seen in a study conducted by Sastri et al in India.^2^

In a study done by Alnusayri on 1716 participants in Saudi Arabia showed similar results of knowledge and attitude difference amongst the general practitioner dentists and specialists.^7^ This in turn potentiates a need for a detailed propostion of basic orthodontic principles in the dental undergraduate course over other detailed teaching of construction of certain appliances. These graduates need to understand the basic diagnosis and referral system in more details for the overall patient welfare.

In contrast to our study, moderately satisfying results were seen in another study done by Niveda S in Chennai on a similar study sample.^3^ Many other studies have also been done on the knowledge of key parameters of malocclusion in many parts of the world.^8,9^ Many of these studies support the fact that dental graduates do not have an ample knowledge of basic malocclusion concept.^10^ Even with the non-orthodontic specialities, though the score was better than general practitioner dentists but it can further be improved by setting up of multi-disciplinary seminars and forums while they undergo education at their post-graduation schools.

A unique study was done in Ireland in which a survey was done among the dental practitioners about the undergraduate orthodontic training that they have had and the extent to which they apply it in their practice. The results showed are in contrast to the present study as the knowledge depicted here in the understanding of similar orthodontic concepts showed a 54% of positive response about the academic knowledge. About 60% of them said they could handle orthodontic emergencies. And 70% aspired to go for higher education in this field.^11^

The present study is just the first step towards a bigger question. The end goal is to make sure the dental graduates have enough knowledge so that a proper referral of such cases can be made to an orthodontist in time. However, the limitation of this study is the geographic area covered and the sample as it was a convenient sample. More studies done in this niche would potentiate the findings and help in acquiring better evidence based conclusion.

## CONCLUSIONS

The present study has shown that the current knowledge and attituide of general practitioner dentists as well as specialties towards the principles and concepts of orthodontic treatment is not satisfactory and that of general practioner dentists is significantly lower than the non-orthodontic specialists. From this knowledge we can emphasis the need for a increased clinically oriented education in the undergraduate courses and a multi-disciplinary inter department seminar presentations and forums set up for the post graduation courses for them to understand the scope of each other's specialties.

## Conflict of Interest

**None.**
